# Supplementary material for: Pyruvate Dehydrogenase Kinase 1 inhibition mediated oxidative phosphorylation enhancement in cartilage promotes osteoarthritis progression
Source: BMC Musculoskelet Disord. 2023 Jul 20;24:597. doi: 10.1186/s12891-023-06585-6 (PMC10357736; doi:10.1186/s12891-023-06585-6)
Supplement: Supplementary file 1 — Additional file 1. [file 12891_2023_6585_MOESM1_ESM.zip › BP.Gsea.1653623667859/GOBP_PEROXISOME_ORGANIZATION.html]

Details for gene set GOBP\_PEROXISOME\_ORGANIZATION[GSEA]

|  || Dataset | 55457\_collapsed\_to\_symbols.cls.cls #OA\_versus\_normal.cls.cls #OA\_versus\_normal\_repos |
| Phenotype | cls.cls#OA\_versus\_normal\_repos |
| Upregulated in class | OA |
| GeneSet | GOBP\_PEROXISOME\_ORGANIZATION |
| Enrichment Score (ES) | 0.49775967 |
| Normalized Enrichment Score (NES) | 1.8255997 |
| Nominal p-value | 0.0 |
| FDR q-value | 0.07087874 |
| FWER p-Value | 0.949 |
Table: GSEA Results Summary

  

Fig 1: Enrichment plot: GOBP\_PEROXISOME\_ORGANIZATION      
 Profile of the Running ES Score & Positions of GeneSet Members on the Rank Ordered List

  

| SYMBOL | TITLE | RANK IN GENE LIST | RANK METRIC SCORE | RUNNING ES | CORE ENRICHMENT || 1 | PEX3 | peroxisomal biogenesis factor 3 [Source:HGNC Symbol;Acc:HGNC:8858] | 11 | 1.178 | 0.0902 | Yes |
| 2 | PEX12 | peroxisomal biogenesis factor 12 [Source:HGNC Symbol;Acc:HGNC:8854] | 74 | 0.956 | 0.1592 | Yes |
| 3 | PEX2 | peroxisomal biogenesis factor 2 [Source:HGNC Symbol;Acc:HGNC:9717] | 94 | 0.921 | 0.2289 | Yes |
| 4 | SCP2 | sterol carrier protein 2 [Source:HGNC Symbol;Acc:HGNC:10606] | 105 | 0.905 | 0.2980 | Yes |
| 5 | FIS1 | "fission, mitochondrial 1 [Source:HGNC Symbol;Acc:HGNC:21689]" | 250 | 0.785 | 0.3475 | Yes |
| 6 | PEX11B | peroxisomal biogenesis factor 11 beta [Source:HGNC Symbol;Acc:HGNC:8853] | 266 | 0.777 | 0.4064 | Yes |
| 7 | PEX7 | peroxisomal biogenesis factor 7 [Source:HGNC Symbol;Acc:HGNC:8860] | 467 | 0.673 | 0.4428 | Yes |
| 8 | MFF | mitochondrial fission factor [Source:HGNC Symbol;Acc:HGNC:24858] | 932 | 0.531 | 0.4479 | Yes |
| 9 | ACOT8 | acyl-CoA thioesterase 8 [Source:HGNC Symbol;Acc:HGNC:15919] | 1088 | 0.500 | 0.4744 | Yes |
| 10 | PEX10 | peroxisomal biogenesis factor 10 [Source:HGNC Symbol;Acc:HGNC:8851] | 1253 | 0.467 | 0.4978 | Yes |
| 11 | PEX19 | peroxisomal biogenesis factor 19 [Source:HGNC Symbol;Acc:HGNC:9713] | 2223 | 0.336 | 0.4484 | No |
| 12 | ABCD2 | ATP binding cassette subfamily D member 2 [Source:HGNC Symbol;Acc:HGNC:66] | 3031 | 0.246 | 0.4048 | No |
| 13 | PEX1 | peroxisomal biogenesis factor 1 [Source:HGNC Symbol;Acc:HGNC:8850] | 3655 | 0.190 | 0.3710 | No |
| 14 | PEX14 | peroxisomal biogenesis factor 14 [Source:HGNC Symbol;Acc:HGNC:8856] | 3985 | 0.157 | 0.3576 | No |
| 15 | PLAAT3 | phospholipase A and acyltransferase 3 [Source:HGNC Symbol;Acc:HGNC:17825] | 4013 | 0.155 | 0.3675 | No |
| 16 | TMEM135 | transmembrane protein 135 [Source:HGNC Symbol;Acc:HGNC:26167] | 5646 | 0.020 | 0.2422 | No |
| 17 | PEX13 | peroxisomal biogenesis factor 13 [Source:HGNC Symbol;Acc:HGNC:8855] | 6228 | -0.023 | 0.1989 | No |
| 18 | PEX6 | peroxisomal biogenesis factor 6 [Source:HGNC Symbol;Acc:HGNC:8859] | 6325 | -0.030 | 0.1937 | No |
| 19 | ZFAND6 | zinc finger AN1-type containing 6 [Source:HGNC Symbol;Acc:HGNC:30164] | 6911 | -0.073 | 0.1539 | No |
| 20 | PEX11A | peroxisomal biogenesis factor 11 alpha [Source:HGNC Symbol;Acc:HGNC:8852] | 7167 | -0.093 | 0.1413 | No |
| 21 | DNM1L | dynamin 1 like [Source:HGNC Symbol;Acc:HGNC:2973] | 8499 | -0.194 | 0.0528 | No |
| 22 | ABCD4 | ATP binding cassette subfamily D member 4 [Source:HGNC Symbol;Acc:HGNC:68] | 8652 | -0.205 | 0.0568 | No |
| 23 | ABCD3 | ATP binding cassette subfamily D member 3 [Source:HGNC Symbol;Acc:HGNC:67] | 8877 | -0.223 | 0.0566 | No |
| 24 | PEX16 | peroxisomal biogenesis factor 16 [Source:HGNC Symbol;Acc:HGNC:8857] | 8921 | -0.226 | 0.0707 | No |
| 25 | ABCD1 | ATP binding cassette subfamily D member 1 [Source:HGNC Symbol;Acc:HGNC:61] | 8930 | -0.226 | 0.0876 | No |
| 26 | RAB8B | "RAB8B, member RAS oncogene family [Source:HGNC Symbol;Acc:HGNC:30273]" | 10223 | -0.343 | 0.0138 | No |
| 27 | PEX26 | peroxisomal biogenesis factor 26 [Source:HGNC Symbol;Acc:HGNC:22965] | 10303 | -0.351 | 0.0347 | No |
| 28 | PEX5 | peroxisomal biogenesis factor 5 [Source:HGNC Symbol;Acc:HGNC:9719] | 10737 | -0.398 | 0.0318 | No |
| 29 | PEX5L | peroxisomal biogenesis factor 5 like [Source:HGNC Symbol;Acc:HGNC:30024] | 10805 | -0.406 | 0.0580 | No |
| 30 | LONP2 | "lon peptidase 2, peroxisomal [Source:HGNC Symbol;Acc:HGNC:20598]" | 11699 | -0.532 | 0.0297 | No |
| 31 | MAVS | mitochondrial antiviral signaling protein [Source:HGNC Symbol;Acc:HGNC:29233] | 12646 | -0.822 | 0.0197 | No |
Table: GSEA details [plain text format]

  

Fig 2: GOBP\_PEROXISOME\_ORGANIZATION      
 Blue-Pink O' Gram in the Space of the Analyzed GeneSet

  

Fig 3: GOBP\_PEROXISOME\_ORGANIZATION: Random ES distribution      
 Gene set null distribution of ES for **GOBP\_PEROXISOME\_ORGANIZATION**
